# Understanding fertility behavior of the Forcibly Displaced Myanmar Nationals in Bangladesh: A qualitative study

**DOI:** 10.1371/journal.pone.0285675

**Published:** 2023-05-30

**Authors:** Md. Anwer Hossain, Mohammad Bellal Hossain

**Affiliations:** Department of Population Sciences, University of Dhaka, Dhaka, Bangladesh; Seisa University, JAPAN

## Abstract

**Introduction:**

Rohingya- the Forcibly Displaced Myanmar Nationals (FDMN)- are largely characterized by a high total fertility rate (TFR) and a low contraceptive prevalence rate. This study aimed to explore the reasons behind their high fertility behavior by utilizing the Theory of Planned Behavior.

**Data and method:**

We adopted a cross-sectional qualitative research approach. Fifteen semi-structured, face-to-face in-depth interviews were conducted with the Rohingya husbands, wives, and community leaders (*Majhi* and Imam/*Khatib*) living in Camps 1 and 2 of Ukhiya Refugee Camp, Cox’s Bazar, Bangladesh. We analyzed the qualitative data using the thematic analysis approach.

**Results:**

The Muslim-majority FDMN predominantly constructed the fertility outcome as the will and order of Allah. On the one hand, the Rohingya parents highlighted various religious, political, economic, and social advantages of having more children, especially sons. On the other hand, beliefs about religious restriction, fear of side effects, and community pressure against contraception sustained the reality of the low contraceptive prevalence rate in the community. Alarmingly, the Rohingya religious leaders and mass people were found highly politically motivated to continue the practice of high fertility with a view to ’*expanding the Rohingya community’* or ’*to increase Muslim soldiers’*, so that they may fight back and take control of their ancestors’ place in Myanmar in the future. Furthermore, these pronatalist attitudes and beliefs translated into high TFR through various high-fertility-supportive social norms and practices widely prevalent in the Rohingya community. These include child marriage, gendered division of labor, women’s subordinate nature, the *Purdah* system, and joint-family members’ support during childbirth and rearing.

**Conclusion:**

Religion, ethnic identity, and the unique political context and experiences of the Rohingya people jointly explain their high fertility behavior. This study warrants the urgency of initiating social and behavior change communication programs to change the religiopolitically-motivated high-fertility notions that prevailed in the Rohingya community.

## Introduction

More than one million Rohingya- the Forcibly Displaced Myanmar Nationals (FDMN) have settled in the refugee camps in Cox’s Bazar, Bangladesh [1]. The existing surveys and demographic profiling studies highlight that high fertility is the prevalent norm among the Muslim-majority Rohingya community, with the widespread practice of child marriage [2–5]. Though there is a scarcity of fertility-related data in this community, the available estimate shows that the total fertility rate (TFR) is 3.8 among the Rohingya [6] which is comparable to other Muslim refugee populations across the world: 3.6 among the Afghan refugees in Iran [7], 3.8 among Syrian refugees in Turkey [8], and 4.0 among the Somalian refugees in Norway [9]. However, worldwide, where Muslims are the majority group in terms of religious affiliation have significantly lower fertility levels, e.g., 1.7 in Iran [10], 2.1 in Maldives, 2.3 in Bangladesh and Turkey, 2.4 in Indonesia, and 2.5 in Morocco [11].

The prevailing high fertility situation is more worrying considering the potential future growth of the Rohingya population as reflected in the age-sex structure of the FDMN; almost 60% of the total population was under the age of 18 years, and about 70% of the Rohingya women of reproductive age (15–49 years) were below 30 years [1, 2]. On the other hand, the proportion of ever-married women was 72.2% among the FDMN, where the mean age at first marriage was 16.8 ± 2.2 years [2]. Along with the favorable age-structure for high fertility, evidence also shows that the Rohingya population is less approving of contraception [2, 3]. While 86.3% of the Rohingya women knew at least one modern method of family planning (FP), and almost half (48.9%) of the currently married Rohingya women knew the place of FP service delivery, the contraceptive prevalence rate (CPR) was found only 33.7% [3]. The CPR among the FDMN is much lower compared to Syrian refugees (52.5%) [12] and Afghan, Sudanese, Somalian, and Saudi Arabian refugees (42%) in Turkey [13]. Similarly, CPRs remain much higher among the major Muslim countries worldwide, e.g., Egypt-58.5%, Bangladesh-61.9%, Morocco-63%, Indonesia-63.6%, and Turkey- 73.5% [11]. In addition to the low use of contraceptives, the available literature shows that child marriage and preference for more children is also the dominant attitude of the Rohingya community [2, 3, 5].

However, given the current state of the FDMN in the settlement camps, their issue of high fertility behavior demands serious concern at least from two perspectives- i) the well-established negative consequences of high childbirths on maternal and child health [14, 15], and ii) multi-dimensional threats and challenges (i.e., human security, tension with the host community, environmental degradation, etc.) of the fast growth of FDMN in Bangladesh [16]. However, despite the importance of the issue, no study was found that directly aimed to understand the context of Rohingya high fertility behavior and explore the reasons behind it using a theoretical framework. Thus, a crucial knowledge gap exists in understanding the context of the Rohingya high fertility behavior. Against this backdrop, the present study aims to provide an in-depth understanding of the context and explore the reasons behind the high fertility behavior of the Rohingya population.

## Methods

### Theoretical orientation of the study

**Fig 1:**
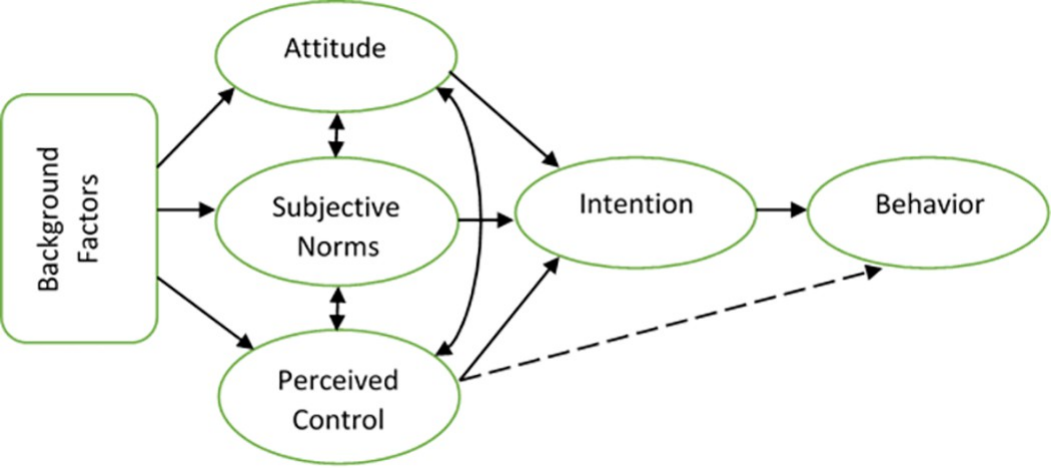
The framework of the theory of planned behavior.

The present study draws upon the social-psychological literature used in major fertility behavior-related studies- the Theory of Planned Behavior (TPB) [17–20] (Fig 1). The TPB argues that human behavior is driven by *intentions*, where intentions are the culmination of a combination of three antecedents, called *belief structure*: (1) attitudes towards the behavior, e.g., perceived costs and benefits of higher/lower number of children; (2) subjective norms surrounding the behavior, e.g., age at marriage, gender role, women’s position in society, etc.; and (3) perceived control over the behavior, i.e., the extent to which fertility is perceived as subject to control by the individual or couple, like through contraception [17–20].

The study’s theoretical orientation is grounded on the TPB because we assumed that the preference for high fertility among Rohingya couples and the low use of contraceptives are associated with the belief structure. Thus, we intended to gain access to the belief structures of Rohingya parents and understand the rationales of their pronatalist norms and practices from their point of view. In addition, the TPB sensitizes users to think about the mechanisms linking ’background factors’ and fertility [20]. In this regard, we also aimed to examine how, on a distant level, various background factors like religion, ethnicity, and social and political considerations had laid the foundations and molded the belief structures of the individuals or couples and ultimately reinforced the practices of high fertility in the Rohingya community.

### Research strategy and design

This study adopted the qualitative research strategy based on the interpretivist epistemology [21], as we aimed to understand the context of high fertility among the FDMN by gaining access to the Rohingya people’s ’common-sense thinking’ about the way they rationalize their high fertility behavior. In addition, the study adopted a cross-sectional research design, as the data were collected at a single time [21].

### Study area and participants

The population of interest was the FDMN, commonly known as the ’Rohingya’. They were residing at several refugee camps in the Ukhiya and Teknaf sub-districts of Cox’s Bazar District, Bangladesh. However, the study was conducted among the Rohingya husbands, wives, and community leaders living in Camps 1 and 2 of the Ukhiya Refugee Camp. These two camps were selected purposively due to their proximity to Cox’s Bazar. The inclusion criteria for the respondents of this study were: 1) the Rohingya parents (either the father or the mother) who had five or more children ever born, or 2) the community leaders (CL)- the *Majhi* (local representative selected by the Bangladesh Army in each settlement camp) or the Imam/*Khatib* (religious leader of the community).

### Sampling procedure and sample size

Data were collected from 15 participants using a purposive sampling technique. Of them, 10 were Rohingya parents (either the father or the mother). The remaining five participants were the local leaders of the Rohingya community: two *Majhi* and three Imam/*Khatib*. It is important to mention that the titles of the local leaders were not mutually exclusive, meaning that among the two *Majhi*, one was also the Imam of his territory, and one of the Imams was also the *Majhi* of his block. This is particularly important, as highlighted in the results section that follows, because of the authoritarian significance these titles have in matters related to fertility. However, the sample size provided sufficient theoretical and data saturation.

### Data collection

Data collection for the study was done in two phases in November 2019. First, qualitative data were collected through the face-to-face in-depth interview (IDI) with the participants using a semi-structured interview guideline (S1 Appendix). The interview guideline was developed based on extensive literature reviews and further refined based on emerging results. Thus, the guideline was used to structure the interviews but allowed for flexibility. For the men participants, the interview duration ranged between 30–45 minutes, while for the women participants, it was 25–35 minutes. The interviews were conducted in the native language of the Rohingya population. Two interviewers (one male and one female) who were fluent speakers of the native language of Cox’s Bazar, Bangladesh, were recruited due to the linguistic and dialectical similarity with the Rohingya populations [3]. The entire interview was audio-recorded with the verbal consent of the participant due to the widespread illiteracy among the FDMN.

### Data analysis

Data analysis was performed based on the interpretative approach in qualitative research methodology [21]. The audio-recorded interviews were transcribed in English, and integrity was checked to ensure accuracy. We adopted a hybrid coding approach that integrated data-driven codes with theory-driven ones [22]. At first, we coded the transcripts using *a priory* coding framework (inductive coding) based on the TPB constructs. All the transcripts were carefully read and re-read by the authors separately. During this process, the first-order themes and sub-themes were noted by each author independently. The two authors then shared the output of emergent codes with each other, which later, through discussion and modification, formed the final coding framework (deductive coding). There were no major disagreements about the identified codes between authors. Two authors then concurrently discussed, crosschecked, and coded the entire data using NVivo 10. We then analyzed the transcripts using the thematic analysis approach to identify relevant themes under the components of the TPB.

The data analysis was an iterative process. As the data were collected in two phases, preliminary raw qualitative data were intermittently analyzed after the first phase to look for emerging themes. The preliminary results were then translated into the interview guideline for the follow-up data collection. In addition, initial results were continuously confirmed or refuted in the field until theoretical and data saturation was reached. The emerging themes under the components of the TPB are identified and written out in the results section. In addition, relevant de-identified quotations of the participants are used to support the arguments from the data. The Standards for Reporting Qualitative Research (SRQR) checklist guided the reporting of the study findings (S2 Appendix).

### Ethical considerations

This study was approved by the Department of Population Sciences, University of Dhaka. However, the study also required site approval. In this regard, the Refugee Relief and Repatriation Commissioner, Bangladesh, and Camp In-Charges of Camps 1 and 2 of the Ukhiya Rohingya Refugee Camps granted permission to conduct this study. The participants of this study were informed about the study, the topic, the type of questions to be asked, and their right to decline to participate or to interrupt the conversation at any time during the interview. Verbal informed consent was obtained from the participants and it was audio-recoded. Before each interview, participants were asked about her/his consent to participate and to let the conversation be audio-recorded upon guaranteeing the participant’s anonymity and confidentiality. People were automatically excluded from the study sample when consent was not obtained. The male interviewer and the first author conducted interviews with the men participants. The female interviewer interviewed the women participants, and the first author provided close supervision. Transcripts and relevant quotations used in the results section are anonymous with codes (M-Man, W-Woman, CL-Community Leader).

## Results

### Background characteristics of the participants

Among the 15 participants, 10 were men (5 fathers and 5 CLs- 2 *Majhi*, 3 Imam/*Khatib*), and the remaining 5 were women (mothers) whose age, level of education, and occupation data are summarized in Table 1. The findings about the men participants’ current and previous occupations (in Myanmar) reveal an important dimension. Most FDMN had experienced a shift in their occupation due to the (forceful) displacement. For example, a farmer had become a day laborer; a day laborer had become jobless, while a businessman, boatman, or driver had no current job (Table 1). However, jobs or professions shifted for all except for the Imams. Individuals, who were Imam in the Rohingya community in Myanmar, were also acting as either Imam or had become *Majhi* of their respective blocks or both together in the settlement camps in Bangladesh. Thus, the authoritarian significance of an Imam, the Muslim religious teacher in the Rohingya community, prevailed well even after the displacement.

**Table 1:** Socio-demographic characteristics of the participants.

ID	Age (years)	Education Level	Current Occupation (Occupation in Myanmar)
M 01	35	No Education	Day Labourer (Driver & Boatman)
M 02	35	7 Class	Day Labourer (Farmer)
M 03	40	No Education	No Job (Day Labourer)
M 04	42	3 Class	No Job (Business of trees & others)
M 05	50	9 Class	*Majhi* (Local Representative/ Member)
W 01	35	No Education	Housewife
W 02	40	No Education	Housewife
W 03	35	No Education	Housewife
W 04	43	No Education	Housewife
W 05	30	No Education	Housewife
CL 01	35	12 Class	Imam (Imam)
CL 02	35	12 Class	Imam (Imam)
CL 03	42	12 Class	*Khatib* (Religious Teacher)
CL 04	45	No Education	Majhi (Land Owner, Business)
CL 05	40	11 Class	*Majhi* (Imam)

Note: M-Man; W-Woman; CL-Community Leader

### Participants’ attitude towards reproduction and fertility intention

Fertility intention refers to the intention of parents whether or not to have children in the future. The Muslim Rohingya population largely perceived the fertility outcome as *"the will/order of Allah"*. Thus, most of the population in normal contexts did not intervene in the "natural process" of reproduction, thereby having more children. Among the 15 study participants, 12 responded that they would have more children in the future, while they already had 3 to 12 children each (Table 2). When asked about their future fertility intention, the common responses were *"it is in the hand of Allah"* and *"if Allah wills"*. On the question of the ideal number of children, the common responses were *"as many children as Allah pleases with"* or "*the more*, *the better"* (Table 2). In the words of two participants-

**Interviewer**: How many children do you intend to take in the future?

**Participant 1**: "*What can I say about it*, *brother*? *It is in the hand of Allah*. *I’ll have as many as Allah gives me*.*"* (M 03, 40 years, Father of 07 children)

**Participant 2**: "*The more Allah gives*. *But*, *I think two more will be good enough*.*"* (W 01, 35 years, Mother of 09 children)

**Table 2:** Number of children, fertility intention, and contraceptive use status of the participants.

ID	NOC ^¥^	Fertility Intention (Ideal number of children)	Contraceptive Use
M 01	05	If Allah wills (7/8 children)	Depo-Provera User
M 02	06	In the Future, Yes (the more, the better)	Implant User
M 03	07*	If Allah wills (as many children as Allah pleases)	No
M 04	09	If Allah wills (the more, the better)	No
M 05	08	Do not want any more children	Depo-Provera User
W 01	09	Two more Children (the more, the better)	No
W 02	04^#^	If Allah wills (the more, the better)	No
W 03	12	Not now, but maybe in future	Depo-Provera User
W 04	06	Do not want anymore	Depo-Provera User
W 05	05	Do not want anymore	No
CL 01	04	If Allah wills (as many children as Allah pleases)	No
CL 02	06	If Allah wills (as many children as Allah pleases)	No
CL 03	05	Not anymore	No
CL 04	09	If Allah wills (as many children as Allah pleases)	Depo-Provera User
CL 05	03*	If Allah wills (the more, the better)	No

Note:

^¥^ Number of Children;

* Their wives were pregnant during the interview and intended to have more children in the future;

^#^ The women aspired to have more children and would not use contraceptives.

Some participants even had reservations about interviewers’ wording to conceptualize fertility, that it- the *child*—is not a matter of ’*taking’*. In the words of the *Khatib* (Religious leader)-

*"No*, *no*! *We don’t ’take’*, *brother*. *Allah gives*. *Can you take a single child if Allah doesn’t give you one*? *No*! *It is the blessing of Allah*. *So*, *the way you’re putting it*, *is wrong*. *Please don’t get hurt by my words*.*"*

(CL 03- *Khatib*)

However, irrespective of the fertility intention of the participants, all of them already had high fertility, ranging from 3 to 12 children each. In the context of contraceptive behavior, women were the primary contraceptive users, and only six wives were using contraceptives (Table 2).

### Reasons for high fertility behavior among the participants

The retrospective reasoning about the high fertility among the participants had brought out some narratives of perceived benefits, advantages, or positive values associated with the higher number of children that constructed the parents’ attitude towards the value of children. We have listed the themes and sub-themes containing the study’s main findings in Table 3, to familiarize the readers with the following details.

**Table 3:** List of themes and sub-themes presented in the result section.

**Theme 1: Attitude toward the value of children** Sub-theme 1.1: Perceived religious benefits Sub-theme 1.2: Political benefits, necessity, and will Sub-theme 1.3: Economic benefits Sub-theme 1.4: Social considerations Sub-theme 1.5: Psychological satisfaction
**Theme 2: Supportive Subjective Norms & Practices** Sub-theme 2.1: Perceived gender role Sub-theme 2.2: *Purdah* and subordinate nature of women Sub-theme 2.3: Support from the joint family members Sub-theme 2.4: High TFR-supportive social practices
**Theme 3: Perceived Control over Fertility** Sub-theme 3.1: Religious restriction and fear of punishment Sub-theme 3.2: Fear of side effects Sub-theme 3.3: Community pressure against contraception

### Theme 1: Attitude toward the value of children

As mentioned earlier- having a higher number of children or a large family was *preferred* and, thereby, was a common occurrence among the study participants. Thus, the question of the perceived benefits of having more children revealed some narratives of how Rohingya parents value a child.

#### Sub-theme 1.1: Perceived religious benefits

Participants universally highlighted various religious benefits of having more children. One participant, who was the *Khatib*, provided a detailed description of how, in general, people of their community perceive a child as a "*divine wealth"* that comes from Allah. In his word:

*"Let me tell you something*. *Allah gives people wealth; it is of two types*. *He gives some people enormous wealth whom we call ’rich people’*. *The other kind of wealth*, *the most precious one*, *is Children*. *Many rich people have much wealth*, *huge in amount*, *but they don’t have any children*, *at best one or two…*. *They might have enormous riches*, *but the door of this blessing- the Children*, *is closed for them*. *But we have that wealth in plenty*, *Alhamdulillah*.*"*

(CL 03- *Khatib*)

The Muslim Rohingya parents mostly perceived a boy or girl child as the "*blessing/gift of Allah"*. They believed that having more children implied being a more blissful parent. Parents also believed that having more children was both ways beneficial from a religious point of view. Because on the one hand, they were increasing the number of members of the *Muslim Ummah* (followers of the Islam religion), which was a good deed in itself. On the other hand, parents are rewarded by Allah for every good deed their children do in this world. For these reasons, parents mostly aspired to give their children proper *Ilm* (Islamic education) in their lifetime. Moreover, the children are largely perceived as "*means of refrainment from punishment in the grave and life hereafter"* for their parents. In the words of participants:

*"In our religion of Islam*, *children are the blessings of Allah*. *The more children you have*, *the more blissful you are*. *You’re increasing the number of Muslims*, *isn’t it a good deed too*?*"*

(M 03, 40 years, no education, Father of 07 Child)

*"They* (children) *will be the means for parents to have refrained from the punishment in the grave*. *If I have one Hafez son*, *it will be a great advantage for me in Akhirat* (Life Here-after)."

(W 02, 40 years, Mother of 04 children).

#### Sub-theme 1.2: Political benefits, necessity, and will

The high fertility behavior among the Rohingya was not in practice solely due to perceived religious benefits. It was also necessary for the Rohingya ethnic minority from a political dimension. The identity crises, fear of persecution, and continued domination for being an ethnic minority in their place of origin (Myanmar) led to strengthening their preference for high fertility, intending to increase their "*own people"* or "*to increase Muslim soldiers"*. Most of the study participants narrated their responses with the notion that having a higher number of children was a political advantage and a necessity for them to sustain the very existence of the "*Rohingya"* identity.

*"In Burma*, *we were small in number in comparison with the Monks*. *They used to torture us in every way possible*. *So*, *we needed to increase our people*.*"*

(M 05, 50 years, Father of 08 children)

*"If we take more children*, *people of our religion will increase*. *Our community will expand*.*"*

(W 01, 35 years, Mother of 09 children)

*"As we are an ethnic minority*, *the intention to increase our population is always there amongst us*. *Because nobody cares about the torture and persecution done to a small population*, *but if we are more in number*, *the world will question*, *’Why do you torture them*? *Give them their rights’*.*"*

(CL 02-Imam)

Above all, an Imam described a striking political will that might remain blended in the prevalent high fertility behavior and in notions of "*increasing own people/Muslim soldiers"* or "*expanding the community"*. He believed that if they continued to take more children, then one day, those children would fight against the oppressors and take control of their ancestor’s place in Myanmar. He also said that this was the main intention of the Rohingya people, and they did not want to stay in Bangladesh forever. In his word:

*"Listen*, *brother*, *Arakan* (Rakhine) *is our country*. *We will not be here* (in Bangladesh) *forever*. *We want to go back to our country*. *If we have more men*, *we will take control of our land one day*. *If the world’s countries don’t help us get back to our country*, *our children will fight and take control of our land*. *That’s why we*, *the Rohingya*, *take as many children as possible*. *This is our number one goal*.*"*

(CL 01- Imam)

However, when parents mentioned this political dimension rationalizing their high fertility behavior, a common occurrence was the preference for sons. In addition, sons were perceived as physically strong and advantageous in feuds and disputes.

#### Sub-theme 1.3: Economic benefits

Rohingya parents associated various economic benefits with high fertility. Some participants perceived having more children, especially sons, was more advantageous regarding their economic contributions to the family. The notion largely echoed in the responses of those participants who were not economically solvent in Myanmar (Table 1).

*"There are some economic benefits too for parents*. *If the sons don’t pursue education*, *they mostly work with their fathers*. *It brings more income to the family*.*"*

(M 02, 35 years, Father of 06 children)

Dowry was a widespread norm during marriages in the Rohingya community. It was also a strategy of capital accumulation by the bridegroom’s family. One participant sarcastically narrated that sons are advantageous because they earn money even through their marriages.

*"Of course*, *having more children is advantageous for parents*. *If somebody has more sons*, *they’ll work and earn more money*. *They can take good care of their parents*. *And when they marry*, *they bring money too* (through dowry). *Ha ha ha*!.*"*

(M 03, No education, Father of 07 children)

#### Sub-theme 1.4: Social considerations

We identified some social considerations that led to the preference of high fertility among the participants. For example, Rohingya mothers preferred more children to secure their old age and mostly perceived their sons as their protectors. Moreover, the experience of persecution and killing of Rohingya men in Myanmar further acted as an effect multiplier to perpetuate the high childbirth norm.

*"In Burma*, *they* [military autocrats] *used to kill men*. *Therefore*, *if a woman has lost her husband*, *her children are her last hope*. *Moreover*, *normally as a mother*, *I expect my sons to take good care of me during my old age*.*"*

(W 04, 43 years, Mother of 06 children)

#### Sub-theme 1.5: Psychological satisfaction

Some mothers also highlighted the psychological dimension of *happiness* from having more children. They mostly related the perceived religious benefits with the mental satisfaction derived from the children. In the words of one mother:

*"If I have 10 children*, *when they call me ’Ma’* (Mother), *all together*, *isn’t it happiness for me*? *All of them will perform Salat* (prayer). *That’s also beneficial because I’ll get the rewards too*.*"*

(W 05, 30 years, Mother of 05 children)

These pronatalist attitudes are further sustained and facilitated by some high-fertility-supportive social norms and practices widely prevalent in the Rohingya community.

### Theme 2: Supportive subjective norms and practices

#### Sub-theme 2.1: Perceived gender roles

The responses to the question "*what is the job of a man and a woman in your community*?*"* depicts a classic example of highly gender-biased established norms prevailing in the Rohingya community. All the participants narrated that men would work outside and earn a livelihood while women would stay inside the houses and perform the role of caregiver to the family members. However, most of the participants- both men and women- also believed that their religion, Islam prescribes this pattern of gender roles too. The religious leaders also promoted this gendered division of labor.

*"In our community*, *women stay inside the house and do all the household work*, *recite Qur’an and care for the children*. *They don’t come out of their houses*. *Men do the work outside and earn livelihoods for the family*. *This is also the way prescribed in our religion*.*"*

(W 05, 30 years, Mother of 05 children)

*They* (women) *will stay inside the house*, *cook meals*, *do household chores*, *and take care of the children*

(CL 01- Imam).

The practice of these biased gender roles seemed so deep-constructed inside the community that women themselves had taken it for granted and became its active promoters. Some mothers strongly believed that giving birth and taking care of the children and other family members were the works of women. They were also reluctant to educate their daughters because, in the end, women were to "*come back to the kitchen and do what they are meant to do"*. Apart from the beliefs of men about biased gender roles, women also believed that staying inside the house is better for them because, in that way, their perceived role and *Ibadat* (religious activities) would not be interrupted.

**Interviewer**: But giving more births means more bleeding, more pain, and increased chances of experiencing other life-threatening complications. Still, why would you want to give birth to more children?

**Participant 1**: "*The losses are mine*. *We all have to die one day*, *isn’t it*? *So*, *I accept this*, *and it is not a problem*.*"* (W 01, 35 years, Mother of 09 children)

**Interviewer**: Can’t you make your daughters educated too?

**Participant 2**: "*What would women do being educated*? *End of the day*, *they’ll have to come back to the kitchen*, *no*?*"* (W 02, 40 years, Mother of 04 children)

*"If we stay inside the house*, *it is better for us… women who work outside can’t perform salat in time*. *So how is this good for them*? *That’s why our religion prohibits women to work outside*.*"*

(W 05, 30 years, Mother of 05 children)

#### Sub-theme 2.2: *Purdah* and subordinate nature of women

While the practice of the society was highly gender-biased, the perceived regulation of *Purdah* and overall patriarchal dominance sustained the subordinate nature of women in the Rohingya community. Moreover, Purdah’s regulation is rooted in religious directives of Islam, and our study population, the Muslim-majority Rohingya, perceived it as a mandatory obligation to follow *Islamic Shariah*. For this reason, to understand the prescription of Islam in matters related to *Purdah*, we sought the knowledge and opinion of the religious teacher of the community- an Imam, in this regard. In his words:

*"All living things are created in pairs*. *Allah has created everything in this fashion*. *If your women come outside*, *and a man sees her and praises her seeing her beauty*, *or your woman does that seeing an unknown man*, *this is a sin*. *Allah says in the holy Qur’an*—*"**Wa-karna fee buyutikunna wa-la tararajna tabarrujal ja-hiliyatil ula* [And stay in your houses, and do not display yourselves like that of the times of ignorance (The Qur’an 33:33)]. *Before the revelation of ayat related to Purdah*, *men and women all used to go to Jihad* (war) *together*. *There was no issue about Purdah then*. *However*, *right after the revelation of this ayat of Purdah*, *Prophet (Sm) mandated all to keep women with Purdah who are mature*. *That’s why it is better for women if they don’t come out of their home"*

(CL 05-Imam)

The religious prescription suggests women not to display themselves. However, this ’not displaying’ does not necessarily imply that women should be confined within the four walls of their houses. Men in the Rohingya community, as evidenced in the statements of men participants and community leaders, had taken the duty ’on their hand’ to fulfill the obligation of *Purdah* for women. For example, an Imam described the strict practice of the *Purdah* system while they were in the Rohingya community in Myanmar.

*"In Myanmar*, *no women could come outside of their house*. *If you came out on the road there*, *you would never see any women*. *If any woman were found*, *she would have a miserable day in her home*. *But coming to Bangladesh*, *you’ll see women everywhere"*.

(CL 01- Imam)

However, the patriarchal dominance was not only found in keeping women inside the house, but it was also more prominent in restricting the voice of women and their decision-making. For example, though three women participants were spontaneously willing to take more children in the future, the other two had no choice or freedom in this regard.

**Interviewer**: So, why don’t you tell your husband that you don’t want anymore (child)? That your health is being compromised?

**Participant**: *"My husband says*, *’you might not need more children*, *but I need more’*.*"* (W 03, 35 years, Mother of 12 children)

**Interviewer**: If we provide contraceptives of your choice, will you use them?

**Participant**: "No. I can’t use them. My husband has forbidden me to use contraceptives. I don’t want to use them too." (W 01, 35 years, Mother of 09 children).

#### Sub-theme 2.3: Support from the joint family members

Another universal finding of the study was that all participants acknowledged that pregnancy is valued in the Rohingya community. The wives used to get assistance and support from their family members and relatives during their pregnancies while in Myanmar. The practice was feasible because people used to live with their relatives in Myanmar "forming a clan", which had a political advantage in times of conflict with military extremists. The support from joint family members certainly acted as a catalyst for having more children. After migrating to Bangladesh, when that norm got disrupted due to the segregated settlement in various camps, it became a matter of concern for the parents. Participants, who had started to use contraceptives coming to Bangladesh, highlighted this issue with special attention.

*"We had a large family*. *With all of our relatives*, *we lived together in Burma*. *They used to help a lot*. *While I was pregnant*, *they didn’t let me do heavy work*. *They cooked meals for me and used to take good care of me*. *Now we have got separated*. *They live in different camps*.*"*

(W 04, 43 years, Mother of 06 children, Depo-Provera User)

#### Sub-theme 2.4: High TFR-supportive social practices

Some prevalent social practices like child marriage and polygamy perpetuated the high fertility behavior, which also presented a clear scenario of lower-status women in the Rohingya community. Some women participants said polygamy was related to men’s desire to have more children, especially sons.

*"Polygamy is widely prevalent here*. *It is a tendency of men*. *If any wife can’t give birth to a child*, *they marry another*. *Again*, *when the wife gives birth to many children*, *they still marry another*. *It continues like this*. *It is permitted in our religion too*. *Our prophet (Sm) had 11 wives and has permitted us to have 4 wives*.*"*

(W 04, 43 years, Mother of 06 children; her father-in-law had 4 wives and 18 children in total)

### Theme 3: Perceived control over fertility

#### Sub-theme 3.1: Religious restriction and fear of punishment

In general, the Rohingya people perceived ’contraception’ as something against the religious directives of Islam. Most study participants said contraception was not permitted in their religion, and therefore, it was an act of *"grave sin"*.

*"If I use contraceptives and stop the childbirth*, *the blessings of Allah will be stopped for me*. *Allah will curse me*. *That’s why I won’t use contraceptives*.*"*

(M 04, 42 years, Father of 09 children)

*"It is said in the Qur’an and Hadith that if parents use Depo* (Depo-Provera to mean contraceptives) *not to have children*, *those children will be born in the field of Qiyamah*. *Then they will become snakes and run after those parents*.*"*

(W 02, 40 years, Mother of 04 children)

The religious leaders also stated their position against the use of contraceptives. They narrated that contraception is not permissible according to the regulations directed in Holy *Qur’an* and the *Hadith*. Therefore, those using contraceptives would receive severe punishment on Judgment Day from Allah. Most importantly, one Imam and one *Khatib* mentioned that the use of contraceptives ’to limit childbirth’ as "*equivalent to killing"* those potential children.

*"If someone uses contraceptives to limit the number of children*, *on the day of judgment*, *he/she will be caught red-handed and thrown to Jahannam* (hell)."

(CL 01- Imam)

**Interviewer**: Huzur, after having three children, if I use contraceptives not to have any more children, how is this equivalent to killing? To kill somebody, s/he must have to be alive in the first place, isn’t it?

**Khatib**: "*By using contraceptives*, *you’re closing the path for a baby to be born*. *You’re restricting the births*. *It is equivalent to killing those potential children*. *Allah says in the holy Qur’an*—*"Wa-e-zal maudatu-suilat*. *Bi-aiyi zambin qutilat* [And when the girls (infant) buried alive [as the pagan Arabs used to do] shall be questioned. For what sin she was killed? (The Qur’an 81: 8,9)]" (CL 03-*Khatib*)

However, the Imams and one *Khatib* also mentioned that there is "*some room for using contraceptives"* in Islamic Shariah. To adequately space two consecutive childbirths, and to give ease to the mothers, the use of contraceptives is permissible in Islam. This notion is particularly important because most of the current contraceptive users among the participants were not using contraceptives ’to put a stop’ to childbearing, but rather ’to put a pause’. In the words of a father:

*"Already we’ve 6 children*. *One daughter died and the rest 5 children are not grown up enough*. *If we have more babies now*, *it will be more problematic*. *It will also be very tough for my wife to maintain the kids*. *So*, *considering all these*, *we decided to use contraceptives* (Implant) *for now*. *Once the children are grown up*, *if we take one or two more by the grace of Allah*, *it won’t be any problem*.*"*

(M 02, 35 years, Father of 06 children)

#### Sub-theme 3.2: Fear of side effects

For some participants fear of the side effects for using contraceptives was the key barrier that was preventing them from uptaking, along with the perceived religious restriction.

*"I used Depo* (Depo-Provera) *in Burma thrice*. *But every time I used the Depo*, *I used to get sick*. *I experienced many problems like pain in my abdomen*, *severe bleeding*, *headache*, *physical weakness etc*. *Then I sought medical treatment and decided not to use it any further*.*"*

(W 01, 35 years, Mother of 09 children)

*"Taking pills causes serious headaches*, *and depo* (Depo-Provera) *causes heavy blood loss*. *These are also prohibited to use*. *Allah gives sin to those who use contraceptives*. *I won’t use contraceptives ever*.*"*

(W 05, 30 years, Mother of 05 children)

#### Sub-theme 3.3: Community pressure against contraception

Apart from the perceived religious restriction and fear of side effects, another striking finding related to the low contraceptive use rate may be attributable to the community pressure against contraception, grounded on the political will "*to expand the Rohingya community"* or "*to increase Muslim soldiers"*. The conversation with an Imam inside a Mosque, in the presence of 12–15 other Rohingya men, clearly indicated the notion of community pressure against the use of contraceptive methods:

**Interviewer**: *Is there anybody in this gathering who uses any contraceptive method*? *Be it a pill*, *Depo-Provera*, *or Implant*. *Anybody*?

**Mass reply**: "*No no"*.

**Participant 1**: "We don’t use them".

**Participant 2**: *"Nobody amongst us uses contraceptives*. *If we hear someone use it*, *we tell him not to use it*. *Why*? *Because our Rasul (Sm) has prohibited it*. *He said*, *’You marry amongst those women who are fertile’”*.

### Theory of planned behavior: How does it explain the high fertility behavior of the FDMN?

As discussed earlier, the TPB argues that individual behavior is driven by intentions, which are a function of three determinants: an individual’s attitude toward behavior, subjective norms, and perceived behavioral control [10]. For this study, we operationalized the TPB framework in line with the fertility behavior and defined the three core components forming the belief structure of individuals, as- 1) attitude towards the value of children, 2) subjective norms, and 3) perceived control over fertility (i.e., contraceptive behavior).

We found that the Rohingya peoples’ beliefs about the consequence of having more children determined their attitude toward the value of children. In this regard, parents highlighted religious, political, and economic benefits that promoted high fertility among the FDMN. On the other hand, high-fertility-related supportive subjective norms, such as child marriage, biased gender roles, *Purdah* and subordinate nature of women, and support from the joint family members during childbirth and rearing, played a role. Finally, beliefs about fertility regulation on account of husband, wife, or community people (e.g., religious leaders) influenced individual’s perceived control over fertility. We found that perceived religious restriction and fear of punishment, fear of side effects, and community pressure against contraception sustained the low contraceptive prevalence rate in the community. These three components—attitude towards the value of children, subjective norms, and perceived control over fertility- determined the intention of the couple to have a higher number of children, ultimately resulting in a higher TFR among the FDMN. On a distant level, various background factors, e.g., religion, ethnic identity, political context, and experiences, laid the foundations and molded the belief structure of the FDMN promoting high fertility.

**Fig 2:**
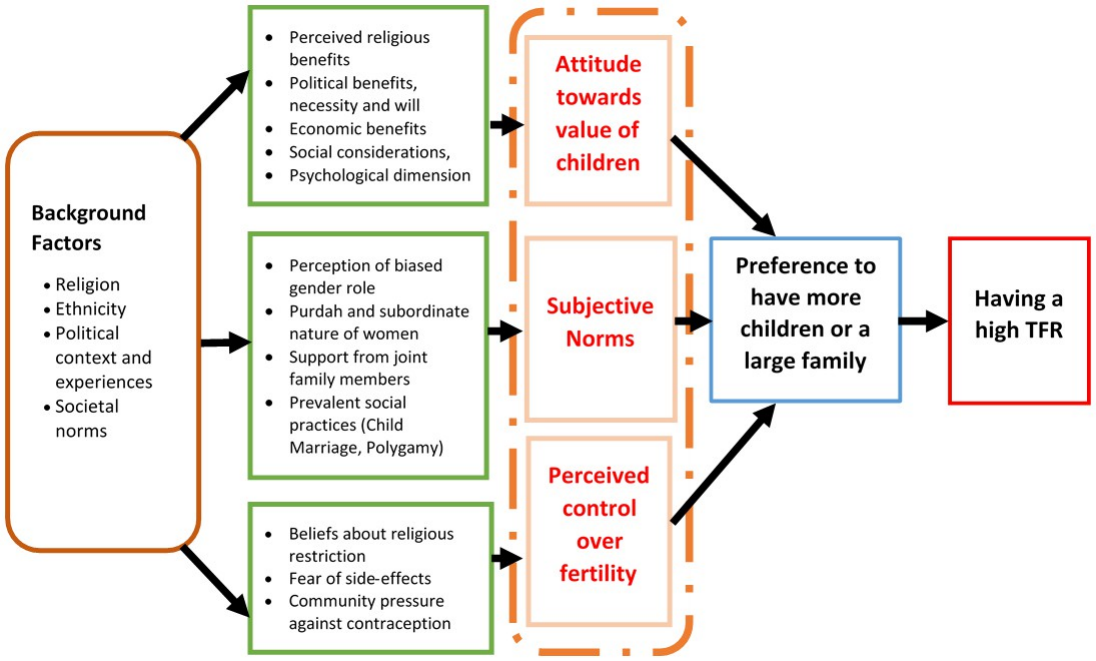
Summary Illustration of the results using the TPB framework.

The findings of the study, as presented throughout the result section, are assimilated and fitted in the following TPB-guided theoretical framework to present that: (1) high TFR among the Rohingya couple is the outcome of planned behavior that results from their high fertility intention, (2) the high-fertility intention is formed and determined by individuals or couples’ belief structure including attitude towards the value of children, supportive subjective norms, and perceived control over fertility through contraception, and (3) individuals’ or couples’ belief structure is influenced by various background factors, e.g., religion, ethnic identity, political context, and experiences, etc (Fig 2).

## Discussions

Rohingya- the Forcibly Displaced Myanmar Nationals—a persecuted Muslim ethnic minority living in refugee camps in Cox’s Bazar, Bangladesh, are largely characterized by a high TFR. The findings of the study suggest that Rohingya high fertility behavior was not merely a case of *blind choice of parents*, as in, for example, the case of the high fertility regime of Bangladesh during the 1970s [23, 24]. Rather having a greater number of children was perceived as religio-politically advantageous by the Rohingya parents, and thereby, it was an intended and rational behavioral outcome from their perspective.

The Muslim-majority Rohingya population predominantly perceived the fertility outcome, i.e., the number of children- as the will and order of Allah. Rohingya parents believed that having more children implies being a more blissful parent. Typical of other fertility studies among Muslim populations as a minority group in their country [25, 26], perceived religious benefits of having more children, on the one hand, and perceived religious restriction about contraception, on the other hand, provided the strongest foundation and justification of the high fertility norm among the Rohingya population. Our findings echoed the religious influence on fertility like that of other Muslim refugee populations across the world, including Somalian refugees living in Oslo, Norway [9], Syrian women in West Bekaa, Lebanon [27], Palestinian women in Gaza, the West Bank, Jordan, and Lebanon [28], and Indochinese refugees in the United States of America [29]. Rohingya parents strongly believed that contraception was not permissible in Islamic Shariah, and thereby, contraceptives would be a grave sin, which echoed a long-standing belief found in the Muslim world [25–26]. The religious authorities- the Imam and the *Khatib* of the Rohingya community- vehemently rejected contraception. They mentioned using contraceptives to limit childbirth as equivalent to ‘*killing of those potential children’* (parents would otherwise have). Egeh et al., (2019) also reported similar views of the Somali religious leaders who argued that using contraceptives with the intention of limiting the number of children was against Islamic values and practice [30]. However, Islamic theologians worldwide cite no clear evidence in Islamic jurisprudence against contraception [31].

However, the pronatalist notions backed by the religion are further strengthened by the unique political reality of the Rohingya ethnic minority. The long history of persecution, domination, and oppression over the Rohingya [32, 33] further rationalized, strengthened, and promoted the high fertility behavior, with religio-political motivations to ‘*expand the Rohingya community’* or ‘*to increase Muslim soldiers’* [34]. Such religio-political pronatalist ideologies had been commonly singled out to account for the persistently high fertility levels among Palestinians [28], “hyper-fertile” Muslims in Ladakh, India [35], Jewish women in Israel [36], and Christian pronatalism in the USA [37]. Apart from perceived religious restriction and fear of side effects, overall community pressure against contraception, grounded on these religio-political motivations, further strengthened and sustained the reality of the low contraceptive prevalence rate among the Rohingya population. Similarly, religious misperceptions, cultural factors, and a lack of knowledge regarding modern contraceptives prevent Somali women and men from accepting and accessing contraceptive health services [30]. Altogether these findings support the minority group status and fertility hypothesis [38] and provide solid empirical evidence in the context of the Rohingya population in the 21^st^ century.

The high-TFR-supportive social norms and practices well established in the Rohingya community also sustained, facilitated, and made the high TFR easy to achieve. The deep-rooted patriarchal systems in the Rohingya community increased the demand for children as they already confined women within the four walls of the houses in the name of *Purdah*, a normative tradition that was widely prevalent in Bangladesh in the 1970-80s as well as in other Muslim traditional societies [39, 40]. The gender division of labor was so deeply constructed within the Rohingya community that women had become active agents to support and promote the biased practice. The majority of the mothers, who were so keen to make their sons *Hafiz* (someone who has memorized Quran) or *Alim* (Islamic scholar), were found reluctant about educating their daughters because of their perceived belief that at the end of the day, daughters would have to “*come back to the kitchen and do what they are meant to do”*. Thus, the girls were married off early, and women had no option but to give birth as soon, with as many children as possible. Hawkey and colleagues [41] found in their study that the phenomena of ‘immediate motherhood’ acted as a social requirement among migrant and refugee women in Australia and Canada. However, having limited opportunities for social status and economic support, a Rohingya mother harnessed self-benefits by giving birth to more children, especially sons. There was also fear of intimate partner abuse and gender-based violence among the less powerful Rohingya women [42]. Moreover, the risk and insecurity that the experience of persecution and killing of Rohingya men in their place of origin posed on women [42] further acted as an effect multiplier to perpetuate the high childbirth norm. Support from the joint family members as ‘co-operative breeders’ [43] and widely prevalent social practices like child marriage and polygamy further eased the reality of Rohingya high fertility to remain functional in their community [4, 44].

We also found that some participants had started using contraceptives after coming to Bangladesh due to inadequate living arrangements and a lack of employment opportunities for Rohingya men outside the settlement camp. The parents of this category firmly believed that contraception is not permitted in Islam with a view to ‘*putting a stop to childbearing’*, rather, it is permitted if the couple use contraceptives to properly space two consecutive childbirth, i.e., ‘to put a pause’. The statements of the religious leaders also supported this belief. However, this particular notion of religious permission for contraception among the Rohingya population needs special attention. Because in the short term, there is a possibility of increasing contraceptive prevalence rate among the Rohingya community, but the perceived benefits from the possible reduction in fertility (due to the poverty-driven contraceptive use or to put a pause in childbearing) are likely to be counter-balanced by the future fertility intention of those short-term contraceptive users.

The study also brings out the striking political will of the religious leaders of the Rohingya community staying in Bangladesh about continuing high population growth to increase the ‘Rohingya’, so that they may fight back and take control of their ancestors’ place in Myanmar. This further echoed the political pronatalism where population numbers were important ideologically and also “can be used as weapons” [45]. However, these religio-political motivations will certainly exacerbate the risks from over-population in an already inadequate living arrangement for the FDMN in the settlement camps. It also warrants a major national security concern for Bangladesh in the long run, especially when the progress of the Rohingya repatriation issue is less obvious [16].

## Strengths and limitations

The study has its significance from multiple dimensions. Firstly, the present study is the first that dedicatedly investigated the fertility behavior of the Rohingya population- one of the most understudied ethnic minorities in the world about whom very little is known. Secondly, the study’s findings have significant implications for the ongoing family planning programs dedicated to the target population. Because the study provides a holistic understanding of the context of high fertility among the FDMN and brings out the in-depth mechanism through which Rohingya parents frame their decision-making favoring high fertility. Thirdly, the study is also unique in operationalizing the Theory of Planned Behaviour and presenting the findings in a simple TPB-guided framework. TPB is most widely used in quantitative, health behavior-related studies though it is open for any creative endeavor [19, 20]. This study is a unique creative utilization of well-established literature like TPB in qualitative research. The study has some limitations too. Firstly, due to time and resource constraints, the study was carried out only in two of the twenty settlement camps- Camp 1 and Camp 2 in Ukhiya, where the FDMN were residing at the time of data collection. Secondly, the interviews with the women participants were comparatively shorter in duration than the men participants. Because of the strict practice of *Purdah*, it was way more challenging to interview eligible women participants, even with the lady interviewer.

## Conclusion

Various surveys and demographic profiling studies characterized the Rohingya- a persecuted Muslim ethnic minority living in refugee camps in Cox’s Bazar, Bangladesh- by a high TFR. The present study aimed to build an in-depth understanding of the context of their high fertility behavior and describe the mechanism using the Theory of Planned Behavior. In short, religion, ethnic identity, and the unique political context and experiences of Rohingya people were found to mold and construct the belief structures of Rohingya parents favoring and sustaining the high fertility norm. Therefore, the ongoing family planning (FP) programs should be scaled up. In this regard, special attention must be paid to the religious leaders of the community- the Imams and the *Khatibs*, because no FP program can yield expected success in keeping them against contraception, given their authoritarian significance in the Rohingya community. Finally, the findings of this study warrant the urgency of implementing a social and behavior change communication program to change the religio-politically motivated high-fertility notion that existed in the Rohingya community.

## Supporting information

S1 Appendix Topic guide for the in-depth interviews. (DOCX)

S2 Appendix Standards for Reporting Qualitative Research (SRQR). (DOC)
