# Supplementary material for: Understanding fertility behavior of the Forcibly Displaced Myanmar Nationals in Bangladesh: A qualitative study
Source: PLoS One. 2023 May 30;18(5):e0285675. doi: 10.1371/journal.pone.0285675 (PMC10228801; doi:10.1371/journal.pone.0285675)
Supplement: S1 Appendix — (DOCX) [file pone.0285675.s001.docx]

**S1 Appendix: Topic Guide for the In-depth Interviews**

| **1. Socio-demographic Information of Participant** |
| --- |
| - Sex - Age - Level of Education - Age at Marriage - Number of Children Ever Born - Children alive and present (by sex) - Children alive but absent (by sex) - Children dead (by sex) - Occupation - In Bangladesh - In Myanmar - Current occupation of the spouse - Number of Siblings alive - Number of Siblings of the spouse - Number of family members - In Myanmar - In Bangladesh |
| **2. Attitude towards Reproduction and Fertility Intention** |
| - How many children do you intend to take in the future? - In your opinion, how many children ideally a family should have? - How do you make the decision about the number of children you want to have?   *Probe: other family members’ influence?*  *Probe: influence of relatives/community norms?* |
| **3. Value of children** |
| - In your opinion, what are the benefits of having more children?   *Probe: Religious?*  *Probe: Political?*  *Probe: Economic?*  *Probe: Social, Psychological?*  *Probe: Other Benefits of a mother/ father/ the family?*   - What are the disadvantages of having few children? - Is there any outspoken preference for boys or girls?   *Probe: advantages of boys? Economic & security concerns? Any disadvantage?*  *Probe: advantages of girls? disadvantages? burden of child marriage?* |
| **4. High-fertility supportive Norms and practices** |
| - What are the works of men and women in your community?   *Probe: Men as bread earners? Women as caregivers?*  *Probe: Why is a such division of roles/labor? What does religion say?*  *Probe: Is there any deviation coming to Bangladesh? How?*   - What is better for women, in terms of roles and works they do? - Husbands’ point of view - Wives' point of view - Attitudes & perceptions of religious authorities - Social Practices - Child Marriage - Polygamy |
| **5. Contraceptive Behavior** |
| - Are you or your wife using any contraceptive methods? Which one? - Have you ever used any contraceptive methods? - What is your opinion about the use of contraceptive methods? - Reasons for not using or low-use contraceptives in the community   *Probe: any religious restriction? Fear of punishment?*  *Probe: fear of side effects?*  *Probe: attitude of other family or community members about contraception?* |
